# Multifractal Dynamic Functional Connectivity in the Resting-State Brain

**DOI:** 10.3389/fphys.2018.01704

**Published:** 2018-11-30

**Authors:** Frigyes Samuel Racz, Orestis Stylianou, Peter Mukli, Andras Eke

**Affiliations:** Department of Physiology, Semmelweis University, Budapest, Hungary

**Keywords:** functional connectivity, dynamic functional connectivity, multifractal analyses, brain, synchronization likelihood (SL), self-organized criticality (SOC), electroencephalography (EEG)

## Abstract

Assessing the functional connectivity (FC) of the brain has proven valuable in enhancing our understanding of brain function. Recent developments in the field demonstrated that FC fluctuates even in the resting state, which has not been taken into account by the widely applied static approaches introduced earlier. In a recent study using functional near-infrared spectroscopy (fNIRS) global dynamic functional connectivity (DFC) has also been found to fluctuate according to scale-free i.e., fractal dynamics evidencing the true multifractal (MF) nature of DFC in the human prefrontal cortex. Expanding on these findings, we performed electroencephalography (EEG) measurements in 14 regions over the whole cortex of 24 healthy, young adult subjects in eyes open (EO) and eyes closed (EC) states. We applied dynamic graph theoretical analysis to capture DFC by computing the pairwise time-dependent synchronization between brain regions and subsequently calculating the following dynamic graph topological measures: Density, Clustering Coefficient, and Efficiency. We characterized the dynamic nature of these global network metrics as well as local individual connections in the networks using focus-based multifractal time series analysis in all traditional EEG frequency bands. Global network topological measures were found fluctuating–albeit at different extent–according to true multifractal nature in all frequency bands. Moreover, the monofractal Hurst exponent was found higher during EC than EO in the alpha and beta bands. Individual connections showed a characteristic topology in their fractal properties, with higher autocorrelation owing to short-distance connections–especially those in the frontal and pre-frontal cortex–while long-distance connections linking the occipital to the frontal and pre-frontal areas expressed lower values. The same topology was found with connection-wise multifractality in all but delta band connections, where the very opposite pattern appeared. This resulted in a positive correlation between global autocorrelation and connection-wise multifractality in the higher frequency bands, while a strong anticorrelation in the delta band. The proposed analytical tools allow for capturing the fine details of functional connectivity dynamics that are evidently present in DFC, with the presented results implying that multifractality is indeed an inherent property of both global and local DFC.

## Introduction

Functions of the brain emerge from dynamic interactions between the elements of its complex neuronal networks (Chialvo, 2010; Werner, 2010). This phenomenon is present across a broad range of spatial scales from the microanatomical level of individual neurons through neuronal cell assemblies to macroanatomical brain regions (Sporns, 2011). Pioneering works by Friston et al. (1993) and Biswal et al. (1995) paved the way to the emergence of a new field of neuroscience aiming at describing brain function through its anatomical and functional connectivity (FC) (Sporns et al., 2005; van den Heuvel and Hulshoff Pol, 2010; Friston, 2011). The key concept underlying the latter is assessing the statistical interdependence of neural activity registered at disparate regions of the brain, as it is assumed to be proportional to the degree of their functional cooperation (Friston et al., 1993). FC studies did not only reveal the existence of several resting-state brain networks such as the default mode network (Raichle et al., 2001; Greicius et al., 2003) or the task-positive network (Fox et al., 2005), but also showed that FC properties responded to changes in physiological conditions e.g., sleep (Horovitz et al., 2009; Liu et al., 2015) or cognitive stimulation (Esposito et al., 2006; Racz et al., 2017). In addition, altered FC was also found in pathological conditions like those in degenerative dementias (Pievani et al., 2011), schizophrenia (Liu et al., 2008), or multiple sclerosis (Cader et al., 2006).

Until recently, most studies typically considered connections in functional networks and thus FC itself, too, as being stationary despite the fact that a dynamic approach might provide a more detailed and more realistic description of brain connectivity (Hutchison et al., 2013a). Indeed it has been shown that FC dynamically fluctuates even in the resting state (Chang and Glover, 2010) and also during task modulation (Sakoglu et al., 2010). Since then, investigating dynamic functional connectivity (DFC) has become one of the most rapidly evolving fields of neuroscience with a steadily expanding body of literature (Hutchison et al., 2013a; Calhoun et al., 2014; Preti et al., 2017).

In FC studies most often functional magnetic resonance imaging (fMRI) is used to monitor brain activity with high spatial resolution and precise anatomical localization (Hutchison et al., 2013a; Preti et al., 2017). Fluctuations in FC are usually captured with a sliding-window (SW) approach, however other approaches such as point process analysis (Tagliazucchi et al., 2012a) or paradigm free mapping (Gaudes et al., 2013) have also been presented. During SW analysis, FC is calculated from a small data segment (i.e., within the actual window), then the window is advanced by a predefined time step and the process is repeated until the whole signal is covered. To assess FC within the actual window, statistical interdependence is usually estimated by bivariate statistical methods as Pearson-correlation (Hutchison et al., 2013b), but multivariate methods such as spatial independent component analysis (Allen et al., 2014) or time-frequency methods (Chang and Glover, 2010) are also often used. DFC is then described through–including but not limited to–properties such as the number of stable global states, their variability, and transition probabilities (Allen et al., 2014; Calhoun et al., 2014; Damaraju et al., 2014). As graph theory provides a useful tool in characterizing complex networks of the brain (Bullmore and Sporns, 2009) along several topological aspects (Rubinov and Sporns, 2010), dynamic graph theoretical analysis is also frequently applied (Tagliazucchi et al., 2012b; Yu et al., 2015; Du et al., 2016; Racz et al., 2018). Finally, some DFC studies focus–instead of on global network topology–only on one or a few individual connections between specific regions (Rack-Gomer and Liu, 2012) or intrinsic connectivity networks (Chang and Glover, 2010; Allen et al., 2014). The fluctuating nature of DFC is then usually captured in measures such as standard deviation (Kucyi and Davis, 2014; Falahpour et al., 2016) or coefficient of variation (Gonzalez-Castillo et al., 2014), however these descriptive measures may be insensitive to finer temporal structuring, which may well be present in DFC.

Although large-scale DFC attracted increasing attention only recently, the dynamic nature of the functional coupling between neuronal cell assemblies had been addressed earlier (Friston, 2000). In fact, functional connectivity for modalities like electroencephalography (EEG) and magnetoencephalography (MEG) was reported having non-linear characteristics (Stam and van Dijk, 2002; Stam et al., 2003). Also, it was shown that several properties of DFC did not have a characteristic time-scale, instead they showed *scale-free* (fractal) dynamics; Gong et al. (2003) presented that fluctuations in phase synchronization between brain regions were scale-free with the characterizing exponent being stable across multiple subjects. Stam and de Bruin (2004) investigated DFC in terms of global synchronization and found that in the alpha and beta bands it scaled with a higher exponent with eyes closed than open. EEG microstates—periods where EEG topography remains constant for 80–120 ms (Lehmann et al., 1987)—also exhibited fractal dynamics as reported by Van de Ville et al. (2010). While these studies evidenced fine, complex temporal structuring present in functional connectivity dynamics both on global (state) and local (individual connection) levels, to the best of our knowledge still only a few studies investigated the scale-free nature of DFC.

Global scale-free (i.e., monofractal) behavior is most commonly characterized by the Hurst exponent (*H*) in the time-, and by the negative power spectral slope (i.e., scaling exponent, β) in the frequency domain (Eke et al., 2000, 2002). *H* and β are inherently interrelated (Eke et al., 2002) as they both characterize the global long-range correlation (LRC) in a signal. This is established by the Wiener-Khinchin theorem stating that the power spectrum is equivalent to the Fourier-transform of the linear autocorrelation function (Kantz and Schreiber, 2004). Describing dynamics through only *H* or β implicitly assumes that frequency components of the power spectrum are independent/random, and information encoded in the phase angles is not considered. According to the definition by Schreiber and Schmitz (2000) this property holds only for linear dynamics. As mentioned above, functional connections in the brain has been shown to be non-linear, which calls for more in-depth analysis techniques capable of providing a detailed-enough description of their dynamic characteristics. At this end, *multifractal* analysis considers scaling as a local instead of a global property of the signal (Mandelbrot, 1986; Tel, 1988; Theiler, 1990), yielding a set of exponents characterizing the scaling in the signal (Kantelhardt et al., 2002; Barunik and Kristoufek, 2010). Moreover, using the method of decomposing a signal into the sign and magnitude time series of its increments (Ashkenazy et al., 2001), it has been shown that multifractal properties of a signal correlated well with its degree of non-linearity (Ashkenazy et al., 2003; Gomez-Extremera et al., 2016; Bernaola-Galvan et al., 2017). Since the seminal work of Ivanov et al. (1999), a diverse set of physiological processes were shown to exhibit multifractal dynamics such as human heart rate variability (Ivanov et al., 2001; Ashkenazy et al., 2003), motor coordination (Ihlen and Vereijken, 2013) or gait dynamics (Ashkenazy et al., 2002). Multifractal analysis of human heartbeat dynamics was also able to capture the separate effects of sympathetic and parasympathetic blockade (Nunes Amaral et al., 2001) as well as reveal the impact of congestive heart failure as the loss of multifractality, substantiating future clinical and diagnostic applications (Ivanov et al., 1999, 2001, 2004). The multifractal nature of neural dynamics was also reported using several different modalities (Shimizu et al., 2004; Wink et al., 2008; Ihlen and Vereijken, 2010), therefore a multifractal approach appears a proper choice when investigating the supposedly rich dynamic properties of functional connectivity.

A recent study using dynamic graph theoretical analysis of multichannel functional near-infrared spectroscopy (fNIRS) data demonstrated that DFC in the human pre-frontal cortex (PFC) expressed multifractal properties (Racz et al., 2018). In this paper we make an attempt on expanding some of the limitations of this previous study: firstly by investigating DFC based on whole-head measurements instead of relying on those only in the PFC, and secondly by considering not only the temporal evolution of global network properties but describing the dynamic fluctuations of individual connections in the network as well. We estimate dynamic functional connectivity based on whole-head EEG measurements using the synchronization likelihood method (Stam and van Dijk, 2002) and apply dynamic graph theoretical analysis. By doing so, we calculate the temporal evolution of three network measures—Density, Clustering Coefficient, and Efficiency—, in order to characterize separate topological aspects of the dynamic networks. Then, both global DFC (as captured in the fluctuations of these network measures) and individual dynamic connections (captured as the fluctuating synchronization levels between regions) are made subject to multifractal time series analysis to reveal their dynamic properties. We performed EEG measurements in eyes open (EO) and eyes closed (EC) states in male and female subjects, which allowed for exploring differences related to state, gender, and network measure. Furthermore, analysis of individual connections between different brain regions allowed us to show if they express not only mono- but indeed multifractal character and also if they show any particular topological pattern regarding their dynamic properties. Our findings imply that multifractal analysis of the dynamics of global functional connectivity as well as that of individual functional connections may provide a valuable tool when extracting information on the temporal structuring of DFC and carry potentials for experimental and/or clinical applications as well.

## Materials and methods

### Participants, experimental protocol and data acquisition

A total of 24 young, healthy volunteers (age: 24.25 ± 2.4 ranging from 20 to 29 years, 12 female) participated in this study. This number of subjects was determined by statistical power analysis of preliminary measurements. The study was approved by the Semmelweis University Regional and Institutional Committee of Science and Research Ethics (ethical approval number: 2017/94) and all subjects provided written informed consent. No participants had reported history of any neurological or psychiatric disorders. Test subjects were instructed not to consume any stimulant (e.g., caffeine) at least 4 h prior to participating and to have at least 6 h of sleep the night before. During the measurement, subjects were seated comfortably in an armchair in a light- and sound-attenuated, electrically sealed room, instructed to remain still, retrain from structured thinking while remaining awake. Resting-state EEG measurements were performed with eyes open (EO) while visually fixating on a dot on a computer screen, followed by another recording session with eyes closed (EC), resulting in four analysis groups: female eyes open, female eyes closed, male eyes open and male eyes closed (F_EO_, F_EC_, M_EO_ and M_EC_, respectively). The recorded signals were visually inspected online, and a session was completed once an artifact-free 305 s record in both EO and EC state have been obtained, which was achieved within 20 min with all subjects.

Measurements were carried out using an Emotiv Epoc+ wireless EEG system (Emotiv Systems Inc., San Francisco, CA, USA), acquiring signals from 14 brain regions according to the 10–20 international system, including AF3, F3, F7 FC5, T7, P7, O1, O2, P8, T8, FC6, F8, F4, and AF4 with additional CMS/DRL reference electrodes at P3 and P4 positions. Data was sampled at 2,048 Hz and internally band-pass filtered between 0.2 and 45 Hz with additional notch filters at 50 and 60 Hz, then down-sampled to an effective temporal resolution of 128 Hz. Electrode impedances were kept under 20 kΩ during signal acquisition. All measurements were performed with maximal contact quality confirmed by the provided Emotiv Xavier TestBench^TM^ software (version 3.1.18).

### EEG data pre-processing

EEG data was pre-processed off-line in Matlab 2012a (The Mathworks, Natick, MA, USA) using the freely available EEGLAB toolbox (Delorme and Makeig, 2004) along with custom scripts and functions. Independent Component Analysis (ICA) was performed (Hyvarinen and Oja, 2000) to remove signal components related to eye movement, blinking, muscle contraction and general noise. These components were identified by their power spectra, visual appearance, and spatial power distribution over the cortex and rejected from the data before performing inverse ICA. Subsequently, the ICA-pruned datasets were band-pass filtered in the traditional frequency bands used in EEG-analysis: δ, 0.5–4 Hz; θ, 4–8 Hz; α, 8–13 Hz; α, 13–30 Hz, and γ, 30–45 Hz according to Stam and de Bruin (2004). All further analyses were also performed on broadband (unfiltered), ICA-pruned signals as well. The first and last 2 s of each measurement segment were rejected, resulting in datasets with length of 38528 data points for both states of every subject.

### Synchronization likelihood

The synchronization likelihood (SL) method (Stam and van Dijk, 2002) was used for pairwise estimation of dynamic functional connectivity. SL identifies non-linear statistical interdependencies between a pair (or in case of global SL, a larger set) of signals. It is by its nature dynamic (i.e., estimates synchronization as a function of time), normalized and seems unaffected by non-stationarities (Stam and van Dijk, 2002). These properties make SL a suitable tool in FC studies using EEG measurements, as EEG signals (and brain activity in general) are often considered non-stationary (Kaplan et al., 2005; Freeman and Quian Quiroga, 2013) and the functional coupling between different neuronal ensembles was confirmed to be non-linear by several studies (Friston, 2000; Stam et al., 2003).

SL measures the general synchronization between discretely sampled processes *x*(*t*) and *y*(*t*), *t* = 1, 2, … *T*. First, the temporal evolution of *x*(*t*) and *y*(*t*) is reconstructed in the state space by temporal embedding (Takens, 1981), where *x*(*t*) and *y*(*t*) is converted into a set of state space vectors *X*(*t*) and *Y*(*t*) as

(1) $$\begin{array}{r} X\left(t\right)=x\left(t,t-m,t-2m,\ldots ,\;t-\left(d-1\right)m\right),\\ Y\left(t\right)=y\left(t,t-m,t-2m,\ldots ,\;t-\left(d-1\right)m\right), \end{array}$$

where *d* is the embedding dimension and *m* is the time lag. Further, let's define the probability for every state space vector *X* (*t*) [and for *Y*(*t*), similarly] that the distance of a randomly selected vector *X* (*t*+*u*) is closer than distance *r*_*x*_(*t*) as

(2) $$\begin{array}{l} C\left(r_{x}\left(t\right),X\right)=\frac{1}{2\left(w_{2}-w_{1}\right)}\sum_{w_{1}<\left|u\right|<w_{2}}\theta \{r_{x}\left(t\right)-\\ \;\left|X\left(t\right)-X\left(t+u\right)\right|\}, \end{array}$$

where *u* is the temporal distance, |·| is the Euclidean norm, θ is the Heaviside step function, *w*_1_ is the Theiler correction for autocorrelation (Theiler, 1986) and *w*_2_ is a window parameter such as *w*_1_≪*w*_2_≪*T*. It should be noted, that *w*_2_ serves as the time window in a SW analysis, and as *u* can be negative as well, the ‘window length’ appears as 2*w*_2_, with the middle 2*w*_1_ segment discarded to avoid effects of autocorrelation. The distance parameters *r*_*x*_(*t*) and *r*_*y*_(*t*) are set for every time point *t* that *C*(*r*_*x*_(*t*), *X*) = *C*(*r*_*y*_(*t*), *Y*) = *p*_*ref*_, with *p*_*ref*_ usually fixed at a value close to 0. Thus, *p*_*ref*_ basically serves as an internal thresholding variable. Finally, the synchronization likelihood at time point *t* is defined as the conditional probability that *Y*(*t*) and *Y*(*t*+*u*) are closer than *r*_*y*_(*t*) given that *X*(*t*) and *X*(*t*+*u*) are closer than *r*_*x*_(*t*) and calculates as

(3) $$\begin{array}{l} SL\left(t\right)=\frac{1}{2p_{ref}\left(w_{2}-w_{1}\right)}\sum_{w_{1}<\left|u\right|<w_{2}}\theta \{r_{x}\left(t\right)-|X\left(t\right)\\ \;-X\left(t+u\right)\left|\right\}\theta \left\{r_{y}\left(t\right)-\left|Y\left(t\right)-Y\left(t+u\right)\right|\right\}. \end{array}$$

*SL*(*t*) is then computed for every time point *t*. Note, that the concept of synchronization likelihood is strongly related to the correlation integral (Grassberger and Procaccia, 1983) and can be considered as a so called “fixed-mass” or “*k*-nearest-neighbor” approach (Theiler, 1990).

In this study, initial parameters of *SL* (*d, m, w*_1_, *w*_2_) were set to fit the filter parameters for each frequency band, according to Montez et al. (2006). Parameter settings for each frequency band and those for broadband EEG data are shown in Table 1.

**Table 1:** Synchronization likelihood parameters for each frequency band.

**Band**	**Range**	***d***	***m***	***w_1_***	***w_2_***
Delta	0.5–4 Hz	25	11	264	1264
Theta	4–8 Hz	7	5	30	1030
Alpha	8–13 Hz	6	3	15	1015
Beta	13–30 Hz	8	1	7	1007
Gamma	30–45 Hz	6	1	5	1005
Broadband	0.5–45 Hz	289	1	288	1288

### Dynamic graph theoretical analysis

Synchronization likelihood was computed on the pruned EEG datasets for all pair-wise combination of the channels, yielding a 14 × 14 weighted synchronization matrix for every time point, in which the connection strength is assumed to be proportional to the level of synchronization between brain regions. Each of these matrices capture the actual topology of the underlying network, and calculating different network measures over them yield Network Metrics Time Series (NMTS) that describe the temporal evolution of network topology. Complex networks have several aspects to their topologies such as modularity or small-worldness (Rubinov and Sporns, 2010). The network is required to contain a sufficiently large number of nodes for network descriptors to make sense, i.e., there is no point in calculating for example the node degree distribution on a network with 14 nodes. It has been demonstrated however, that global network measures Density (D), Clustering Coefficient (C) and Efficiency (E) can be used effectively to describe and capture significant topological differences in smaller networks (Racz et al., 2017). We used the weighted formulas to calculate the aforementioned network measures. Weighted Density (often termed also Connectivity Strength) is the fraction of overall connectivity strength present to the maximal possible connection strength in a network (Rubinov and Sporns, 2010) and calculates as

(4) $$\begin{array}{r} D^{W}=\frac{1}{n\left(n-1\right)}\overset{n-1}{\underset{i=1}{\sum }}\overset{n}{\underset{j=i+1}{\sum }}c_{ij}, \end{array}$$

where *n* is the total number of nodes, and *c*_*ij*_ is the connection strength–in this case, *SL*(*t*) for every *t*–between nodes *i* and *j*. Density is a general measure of “wiring cost” of a network, and is also equal to the average normalized node degree (Rubinov and Sporns, 2010). The Clustering Coefficient of an individual node denotes the fraction of the existing triangles to the maximal possible number of triangles around a node (Rubinov and Sporns, 2010) and was generalized to weighted networks by Onnela et al. (2005). Clustering Coefficient of node *i* calculates as

(5) $$\begin{array}{r} C_{i}^{W}=\frac{2}{k\left(k-1\right)}\overset{n}{\underset{j,h}{\sum }}{\tilde{w}}_{ij}{\tilde{w}}_{ih}{\tilde{w}}_{jh}, \end{array}$$

where *k* is the number of edges connected to node *i* and ${\tilde{w}}_{ih}$ is the weight of the edge between nodes *i* and *j*. Edge weights are normalized by the largest weight present in the network, therefore on binary networks the formula returns with the original definition, that is also equivalent to the fraction of a node's neighbors that are also neighbors of each other (Watts and Strogatz, 1998). The global Clustering Coefficient is the average taken over individual nodes. C is the most general measure of network segregation and related to “local” information flow in the network (Rubinov and Sporns, 2010). Finally, Efficiency is a global parameter capturing network integration, and is often associated with the speed of information processing in a complex system (Rubinov and Sporns, 2010). It is the average inverse shortest path length between all nodes of a network and computes according to

(6) $$\begin{array}{r} E^{W}=\frac{1}{n}\overset{n}{\underset{i=1}{\sum }}\overset{n}{\underset{j=1,i\neq j}{\sum }}\frac{{\left(d_{ij}^{W}\right)}^{-1}}{n-1}\;, \end{array}$$

where $d_{ij}^{W}$ is the length of the shortest weighted path between nodes *i* and *j* (Latora and Marchiori, 2001). It is strongly related to the average shortest path length, however often considered as being a superior measure to the latter in describing network integration (Achard and Bullmore, 2007). Efficiency is related to the “global” information flow in the network (Rubinov and Sporns, 2010). For the sake of simplicity, in the following we will drop the superscripts “*w”* and will refer by D, C, and E to their weighted forms, respectively.

Since SL has an internal step of thresholding, to avoid acquiring an overwhelming amount of results we decided not to use any additional threshold values, as in a previous study (Racz et al., 2018) the dynamic properties of DFC did not show any specific relation to the value of threshold. We calculated the time evolution of Density, Clustering Coefficient, and Efficiency [D(*t*), C(*t*), and E(*t*), respectively] for every subject both in EO and EC states. For the calculations of D, C, and E we used functions of the Brain Connectivity Toolbox by Rubinov and Sporns (2010).

### Focus-based multifractal signal summation conversion (FMF-SSC)

**Figure 1:**
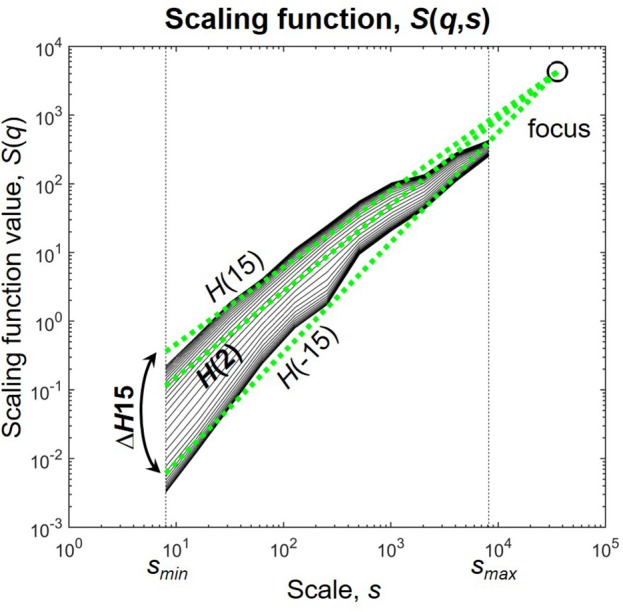
The scaling function. *H*(2) characterizes the global i.e., monofractal scaling, while the difference between *H*(−15) and *H*(15) termed Δ*H*15 captures the degree of multifractality. The focus is used as a reference point for linear regression. Scaling function of D*(t)* of subject male 4 during eyes closed is used for illustration.

Multifractal analysis, instead of a single scaling exponent yields a set of scaling exponents, each describing scaling in fluctuations of different sizes in the signal (Kantelhardt et al., 2002). This can be achieved by characterizing scaling at several statistical moments *q*, where small fluctuations are amplified by the negative, while large fluctuations by the positive moments (Barunik and Kristoufek, 2010; Ihlen, 2013). We used the multifractal generalization of the Signal Summation Conversion (SSC) method (Eke et al., 2000; Mukli et al., 2015) to extensively characterize the power-law scaling of the NMTSs and the SL(*t*)s of individual connections. In SSC, the signal is cumulatively summed and standard deviation σ is calculated at different window sizes ranging from a minimal to a maximal scale (*s*_*min*_ and *s*_*max*_, respectively). Within each window the local linear trend is removed before calculation to diminish effects of non-stationarity. The power-law dependence of σ on *s* is captured in the Hurst exponent *H* according to σ(*s*)∝*s*^*H*^ (Eke et al., 2000). The multifractal generalization of SSC (MF-SSC) consists of repeating the steps of the analysis at different statistical moments *q*, yielding the unified scaling function *S*(*q*,*s*) (Figure 1) (Mukli et al., 2015)

(7) $$\begin{array}{r} S\left(q,s\right)={\left\{\frac{1}{N_{s}}\overset{N_{s}}{\underset{\upsilon =1}{\sum }}\sigma {\left(\upsilon ,s\right)}^{q}\right\}}^{\frac{1}{q}}, \end{array}$$

where *N*_*s*_ is the number of non-overlapping time windows at scale *s*, and υ is the index of the actual window of calculation. As σ is now dependent on *s* and *q* as well, their relationship is established via the generalized Hurst exponent *H*(*q*) according to σ(*q, s*)∝*s*^*H*(*q*)^ and can be acquired by linear regression on the values of *S*(*q*,*s*) (Barunik and Kristoufek, 2010). On empirical signals with finite length the regressed functions converge in one specific point termed *focus* (Mukli et al., 2015). This can be simply shown by replacing *s* in *S*(*q*,*s*) with the total length of the signal as it results in the disappearance of the sum and therefore *q* from equation (7). The focus therefore is used as a reference point during regression, which renders the multifractal analysis of empirical time series very robust and prevents it from ending up with “inverted” or corrupted multifractal spectra (Mukli et al., 2015). Note that this way of handling of empirical signals is not unique to SSC, but can be applied also to other MF methods such as Detrended Fluctuation Analysis or Wavelet Leader methods yielding their focus-based variants (Mukli et al., 2015).

The NMTSs and SL(*t*)s were analyzed by FMF-SSC with the following input parameters as suggested in Mukli et al. (2015): *s*_*min*_ = 8 with *s* increasing dyadically until *s*_*max*_ = 8192 and *q* ranging from −15 to +15 with unit increments. The scaling range was defined between scales 2^3^ and N/4, as scaling function values below and over these scales become statistically unreliable (Cannon et al., 1997; Gulich and Zunino, 2014). Scaling windows based on a dyadic scale provide efficient computation. The range of *q* was selected based on Grech and Pamula (2012), Mukli et al. (2015), and Nagy et al. (2017). Global (i.e., monofractal) scaling associated with the long-term memory of the signal was captured in *H*(2), and the degree of multifractality was described by Δ*H*15 = *H*(−15)–*H*(15), as a measure of how much the scaling is different for small and large fluctuations (Figure 1). Note, that Δ*H*15 captures the distribution of local scaling in a signal equivalently with the often used multifractal/singularity spectrum that can be acquired from *S*(*q*,*s*) by Legendre-transformation (Frisch and Parisi, 1985; Chhabra et al., 1989).

### Surrogate data testing

Multifractal scaling in a time series can appear as a consequence of different long-range correlations present in the signal, however multifractality can also originate in the heavy-tailed probability distribution of signal values without any correlations whatsoever (Ivanov et al., 1999; Kantelhardt et al., 2002). Also, the finite size and/or simple constant linear autocorrelation of the signal can also produce a so-called “multifractal background noise” (Grech and Pamula, 2012), that has to be distinguished from true multifractality. Thus, a proper surrogate data testing is indispensable when analyzing empirical signals. Therefore, all time series (NMTSs and SL(*t*)s) underwent steps of thorough tests to verify if they indeed showed true multifractality. In all of these steps, *n* = 39 surrogate datasets of equal length were generated with the null hypothesis that the investigated time series cannot be distinguished from its surrogates based on the discriminating statistical measure (Theiler et al., 1992). The null hypothesis was rejected if the discriminating statistic was found outside the mean ± 2σ range calculated from the surrogate datasets, that with *n* = 39 surrogate datasets corresponds to a 95% confidence level (Theiler et al., 1992; Kaplan and Glass, 1995).

First, we tested the presence of *global power-law scaling*. This can best be done in the frequency domain, as a signal with global power-law scaling also has a corresponding $\frac{1}{f^{\beta }}$-like spectrum (Eke et al., 2000). Hence, for every time series surrogate datasets with equal β were generated with the spectral synthesis method (Saupe, 1988), and the Kolmogorov-Smirnov distances were calculated to estimate the goodness-of-fit (GoF) of the power-law function. A time series was considered scale-free, if its GoF to a power-law function was within the mean ± 2 σ range of those calculated from surrogate data with known (identical) power-law spectra (Clauset et al., 2009; He, 2011).

Second, we tested the presence of *distribution-related multifractality* by randomly shuffling the values of the time series (Ivanov et al., 1999; Kantelhardt et al., 2002). Shuffling destroys all correlations and reduces the signal to pure random noise but has no effect on the distribution of values. Consequently, if shuffling renders *H*(*q*) ≈ 0.5 (i.e., white noise) for all *q* the case of distribution-related multifractality can be excluded, otherwise presence of scaling (at least partly) could be attributed to a power-law type distribution of signal values.

Finally, we tested if the observed multifractality resulted from the presence of different *long-range correlations*. For this purpose, surrogate datasets were generated by Fourier transforming the signal with Fast Fourier Transformation, randomizing its phases and then performing inverse Fourier transformation (Theiler et al., 1992). Phase randomization leaves the amplitudes and therefore the power spectrum (hence, linear autocorrelation) unaffected (Kantz and Schreiber, 2004), while destroying all non-linear correlations in the signal (Schreiber and Schmitz, 2000). Hence it yields surrogate datasets with equal *H*(2) i.e., *monofractal* characteristic preserved (Eke et al., 2000). As the resulting time series are monofractal signals, this step simultaneously tests for multifractal background noise/true multifractality (Grech and Pamula, 2012) and the presence of non-linearity (Ivanov et al., 1999). Multifractality was considered true and as a sign of non-linear dynamics, if the Δ*H*15 value of a signal was significantly larger than those of surrogate datasets. In the following, we will refer those processes passing all tests as *true multifractals*.

### Analysis strategy

**Figure 2:**
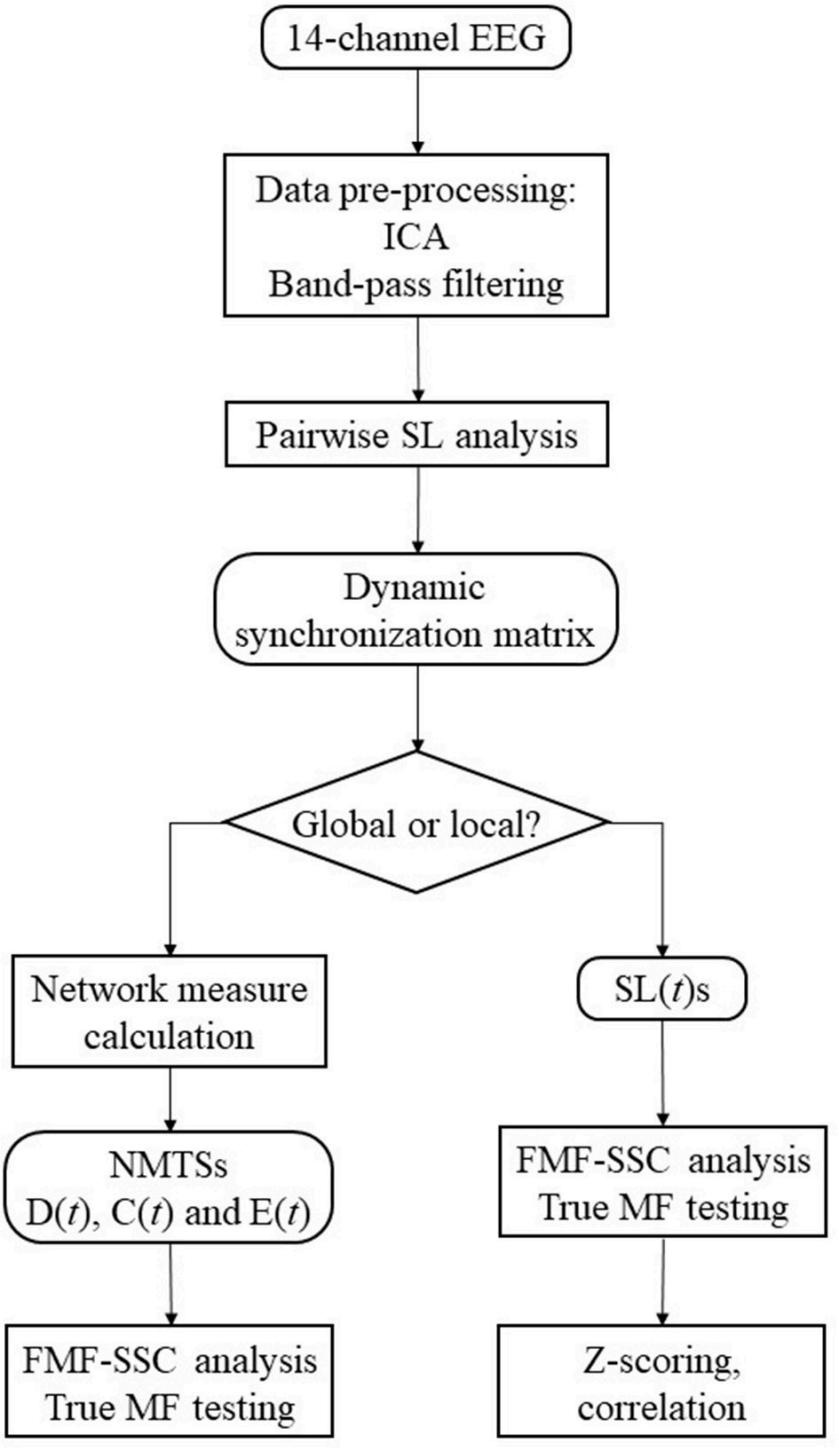
Steps of the analysis procedure.

The flowchart of the analysis steps is shown as a summary of the methods on Figure 2. The first aim of this study was to show if global DFC when investigated by EEG show multifractal properties. For this purpose, the acquired D(*t*), C(*t*), and E(*t*) time series were made subject to surrogate data tests for power-law scaling, long-range autocorrelation and true multifractality as described above, and the fraction of subjects passing each test were computed for all measures. To explore the effect of gender (F and M), network measure (D, C, and E), and state (EO and EC) on the MF properties of global DFC, two-way repeated measures ANOVA tests were performed for each frequency band separately, with gender as grouping variable, while state and network measure as repeated measure factors. Bonferroni *post-hoc* tests were performed to identify significant differences between interactions (in the following marked by ×). Results will be presented in the following manner: for each frequency band, we present the significant differences considering the effect of gender, measure and state separately, along with interaction effects of gender × measure, gender × state and measure × state. When they bear significance, results from the *post-hoc* tests are also discussed in detail.

Our second aim was to show if dynamic functional connections themselves—captured in SL(*t*) between different locations—exhibited multifractal scaling. Therefore, all SL(*t*)s were also processed by the same testing framework as the NMTSs. Fractions of edges in the network passing each test were calculated on the subject level and were averaged combining all four groups (F_EO_, F_EC_, M_EO_, and M_EC_) to obtain a general picture on the presence of true multifractality in functional connections. Subsequently, to reveal if functional connections express any particular topology in regards of their mono- and multifractal properties, *H*(2) and Δ*H*15 values of the connections were standardized (z-scored) on the subject level and averaged across subjects within all analysis groups collectively. This resulted in group-averaged networks where edge weights represent their corresponding z-scored values [z(*H*(2)) and z(Δ*H*15), respectively]. Also, to investigate the possible correlation between the long-term memory and the degree of multifractality of connections, the Pearson coefficient (*r*) was calculated between the group averaged z(*H*(2)) and z(Δ*H*15) values. In order to distinguish between a true correlation effect and pure coincidence, *r* was calculated on the shuffled data as well when *n* = 39 spatial surrogates (Aaria et al., 2013) were generated by randomly shuffling the z(*H*(2)) and z(Δ*H*15) values [thus destroying possible correlation between *H*(2) and Δ*H*15]. Again, if the *r* of original values was outside the mean ± 2 σ range acquired from surrogate data, we regarded the correlation between *H*(2) and Δ*H*15 as significant.

It should be noted, that multifractality of individual connections were investigated in each group (F_EO_, F_EC_, M_EO_, and M_EC_) individually as well, however as results were comparable between analysis groups, for the sake of simplicity we decided to present the results of all groups combined. All statistical analyses were carried out in StatSoft Statistica 13.2.

## Results

### Testing for true multifractality

Results for surrogate data testing of NMTSs (including all groups and states) are shown in Table 2. In the vast majority of the cases, D(*t*), C(*t*), and E(*t*) were proven to have broadband power-law spectra, qualifying them as scale-free (fractal) processes. Shuffling reduced *H*(*q*) approximately to 0.5 in all cases, proving again the presence of long-range correlations in the signals, while also excluding the contribution of distribution-related multifractality. True multifractality was also present in network dynamics in most of the cases for all frequency bands as well for broadband EEG data (Table 2).

**Table 2:** Fraction of NMTSs passing each surrogate data test.

	**Power-law**			**Shuffling**			**Phase randomization**		
**Band**	**D (%)**	**C (%)**	**E (%)**	**D (%)**	**C (%)**	**E (%)**	**D (%)**	**C (%)**	**E (%)**
Delta	97.9	97.9	100	100	100	100	100	100	91.7
Theta	97.9	100	95.8	100	100	100	87.5	93.8	85.4
Alpha	97.9	95.8	100	100	100	100	97.9	93.8	95.8
Beta	100	100	97.9	100	100	100	89.6	89.6	89.6
Gamma	100	100	100	100	100	100	91.7	93.8	95.8
Broadband	97.9	100	100	100	100	100	91.7	100	91.7

Similar results were obtained when investigating the power-law scaling and autocorrelation properties of individual connections in the dynamic functional networks on the subject level (Table 3). In that most connections in the networks yielded power-law spectra, and all connections contained LRCs while distribution-related multifractality could be excluded. Although the fraction of true multifractal connections were generally high in the delta, theta and alpha bands, slightly lower values were found in functional networks of the higher (beta and gamma) frequency bands with a slightly lower amount of connections passing the true multifractality test in broadband EEG networks. Nevertheless, these results compare well with those obtained from global network dynamics (Table 2) implying that the multifractal nature of global network dynamics emerges from multifractally fluctuating individual connection strengths in the network. Note that values in Table 3 refer to the fraction of connections in the functional networks of each subject, thus they are presented as mean ± σ across subjects (combining all four groups).

**Table 3:** Fraction of functional connections in the network passing each test.

**Band**	**Power-law (%)**	**Shuffling (%)**	**Phase randomization (%)**
Delta	99.13 ± 9.29	100	99.47 ± 7.24
Theta	98.92 ± 10.32	100	93.77 ± 24.17
Alpha	98.86 ± 10.64	100	97.09 ± 16.80
Beta	98.99 ± 9.99	100	76.53 ± 42.38
Gamma	98.72 ± 11.25	100	81.87 ± 38.53
Broadband	98.28 ± 12.99	100	89.33 ± 30.87

### Multifractal nature of global DFC

**Figure 3:**
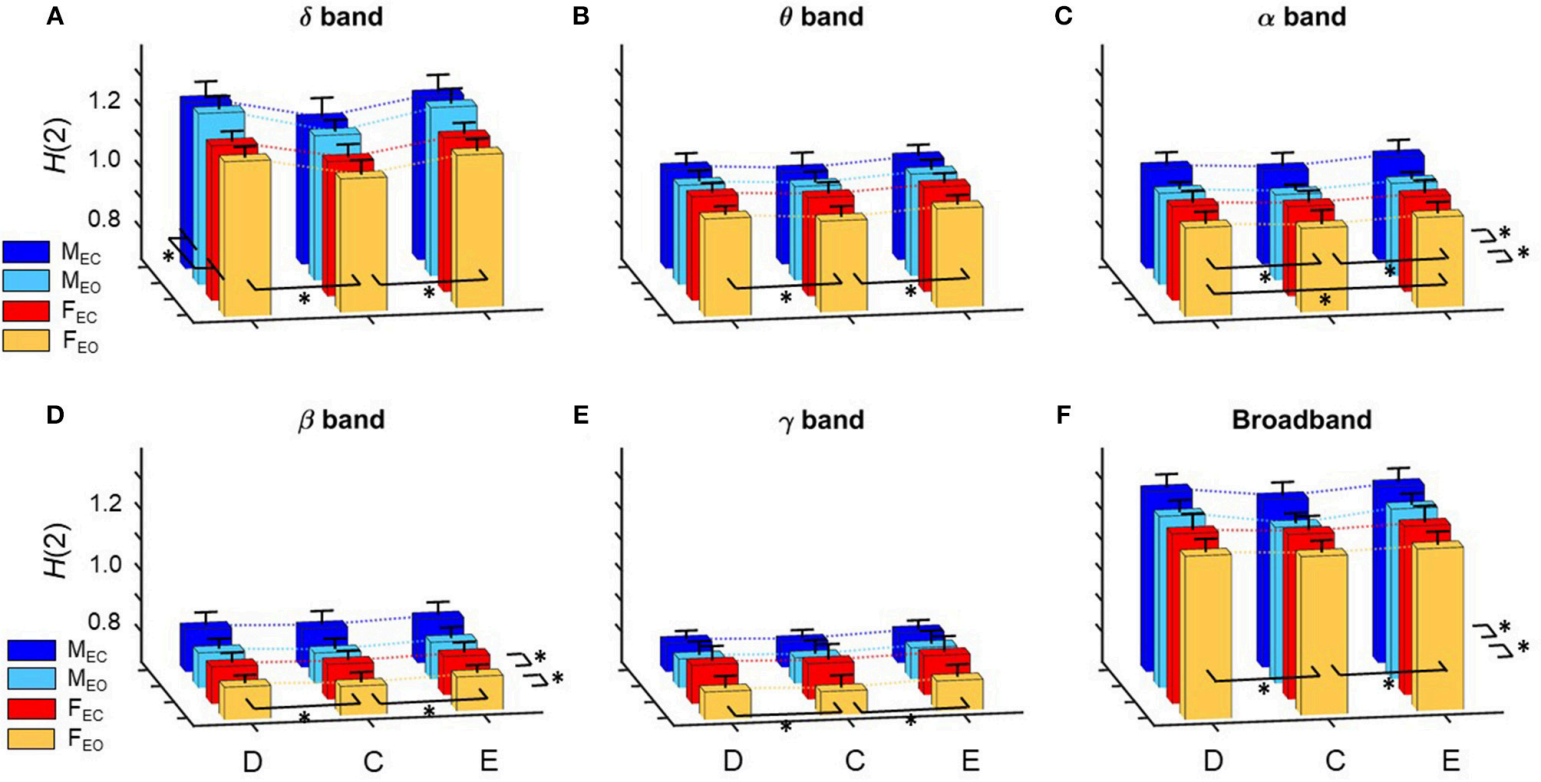
Summary of results regarding *H*(2) of global network metrics for the delta **(A)**, theta **(B)**, alpha **(C)**, beta **(D)**, and gamma **(E)** bands, as well as for broadband EEG **(F)**. Dotted lines connect the means of the corresponding analysis groups. Significant differences are marked by ^*^ for effects of gender, measure and state (displayed on the left, in front and right of each subplot, respectively). Note, that significant differences of interaction effects are not marked.

**Figure 4:**
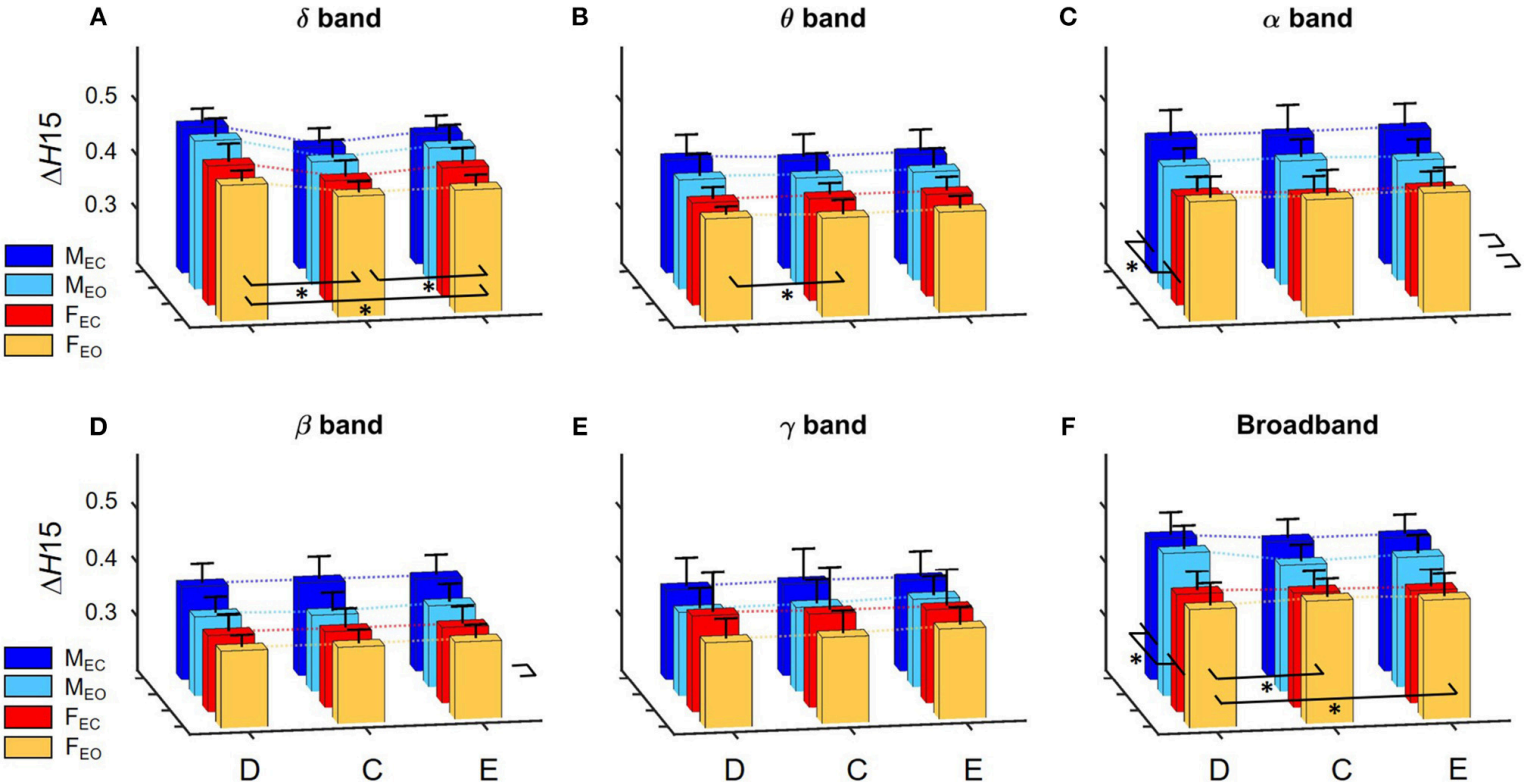
Summary of results regarding Δ*H*15 of global network metrics in for the delta **(A)**, theta **(B)**, alpha **(C)**, beta **(D)**, and gamma **(E)** bands, as well as for broadband EEG **(F)**. Dotted lines connect the means of the corresponding analysis groups. Significant differences are marked by ^*^ for effects of gender and measure (displayed on the left and in front of each subgraph, respectively). Again, significant differences of interaction effects are not marked.

In the following, for the *H*(2) of D(*t*), C(*t*), and E(*t*) we will use the abbreviations *H*_D_(2), *H*_C_(2), and *H*_E_(2), respectively. Similarly, Δ*H*15 of D(*t*), C(*t*), and E(*t*) will be referred to as Δ*H*_D_15, Δ*H*_C_15, and Δ*H*_E_15. Summary of the results regarding *H*(2) of global DFC is shown in Figure 3, while those of Δ*H*15 are presented on Figure 4.

A prominent measure-related effect appeared in *H*(2) which was present in all frequency bands, as *H*_C_(2) values were found significantly lower than those of *H*_D_(2) and *H*_E_(2). The same difference was found regarding Δ*H*15 (Δ*H*_D_15 > Δ*H*_C_15 < Δ*H*_E_15), however in the delta band, only. Moreover, state had a significant effect in the alpha and beta bands as *H*(2) values increased during EC compared to EO condition. Gender related effects were found sparsely with a tendency of higher *H*(2) and Δ*H*15 values in males. In the following, we elaborate on these results for every frequency band separately.

#### Delta band

*H*(2) values were found significantly higher in the male groups (main effect of gender, *p* = 0.023). The three network measures also showed significant difference with *H*_C_(2) being lower than those of *H*_D_(2) and *H*_E_(2) (main effect of measure, *p* < 0.0001). Interaction of measure × gender and Bonferroni *post hoc* tests showed that these differences occurred in both genders (Figure 3A).

Measure had a significant main effect on Δ*H*15 as well (*p* < 0.0001). The measure × gender interaction revealed, that in females Δ*H*_D_15 was significantly larger than Δ*H*_C_15 andΔ*H*_E_15 with no difference between the latter two, while in males Δ*H*_E_15 was also larger than Δ*H*_C_15 (*p* < 0.01 in all cases). Neither gender nor state had significant effect on Δ*H*15 in the delta band (Figure 4A).

#### Theta band

Measure had a similar main effect on *H*(2) as in the delta band [*H*_D_(2) > *H*_C_(2) < *H*_E_(2), *p* < 0.001 in all cases], however neither gender- nor state-related differences were found (Figure 3B).

Regarding Δ*H*15, only measure-related differences were found (main effect of measure, *p* = 0.026). Bonferroni *post hoc* test indicated that Δ*H*_D_15 was higher than Δ*H*_C_15, however the interaction measure × gender showed that this was not significant in neither the male or female groups individually (Figure 4B).

#### Alpha band

The previously observed difference in measure regarding *H*(2) was also found in the alpha band [*H*_D_(2) > *H*_C_(2) < *H*_E_(2), *p* < 0.001 in all cases], however additionally in males *H*_E_(2) was slightly higher than *H*_D_(2) and *H*_C_(2) (*p* = 0.043). Also, a significant difference related to state was revealed with *H*(2) being higher in EC than in EO state (*p* = 0.035). Interaction of measure × state verified that this difference was present in all network measures (Figure 3C).

Larger Δ*H*15 values were found in the male groups (main effect of gender, *p* = 0.014). Interestingly, the interaction gender × state revealed a trend in which state had the opposite effect in the two genders, as Δ*H*15 increased in males while decreased it in females during EC condition (*p* = 0.066) (Figure 4C).

#### Beta band

The main effect of measure was found significant (*p* < 0.0001) with Bonferroni *post hoc* test revealing *H*_C_(2) being smaller than *H*_D_(2) and *H*_E_(2) (*p* < 0.001 in all cases). Moreover, similarly to the alpha band, state had a significant effect in increasing *H*(2) when transitioning from EO to EC (*p* < 0.001) (Figure 3D).

Δ*H*_D_15, Δ*H*_C_15, andΔ*H*_E_15 were found increased in EC state, although the main effect of state was not significant (*p* = 0.136). The interaction of gender × state revealed that this increase was only present in the male group, while Δ*H*15 values remained unchanged in females, although yet again only in tendency (*p* = 0.093) (Figure 4D).

#### Gamma band

In *H*(2), only measure-related differences appeared significant, with a similar tendency as in the alpha band, with *H*_C_(2) being lower than *H*_D_(2) and *H*_E_(2), and in males *H*_E_(2) also being higher than *H*_D_(2) too (*p* < 0.001 in all cases) (Figure 3E). No differences were found regarding Δ*H*15 (Figure 4E).

#### Broadband EEG

Measure related differences were found (main effect of measure, *p* < 0.0001) with Bonferroni *post hoc* test confirming the same tendency as in most previous cases [*H*_D_(2) > *H*_C_(2) < *H*_E_(2), *p* < 0.01]. Also, higher *H*(2) values were found in EC (main effect of state, *p* = 0.014) (Figure 3F).

The main effect of gender on Δ*H*15 appeared significant (*p* = 0.012) with higher values in the male groups. Measure also had a significant effect on Δ*H*15 (*p* = 0.0006) however the measure × gender interaction and Bonferroni *post hoc* test revealed that significant differences only occurred in the male group, with Δ*H*_D_15 being significantly higher than Δ*H*_C_15 and Δ*H*_E_15 (*p* < 0.001 in both cases) (Figure 4F).

In order to keep the statistical analysis simple, frequency band was not included as a factor in the statistical analysis, however it can be clearly seen on Figure 3 that *H*(2) values in the delta and alpha band are considerably higher than those of the theta, beta, and gamma bands, with *H*(2) of unfiltered signals being approximately in between. Figure 4 shows that Δ*H*15 values were the highest in the delta-, alpha-, and broad-band EEG, with slightly lower values in the theta, beta, and gamma bands.

### Individual connection dynamics

#### Long-range correlation–*H*(2)

**Figure 5:**
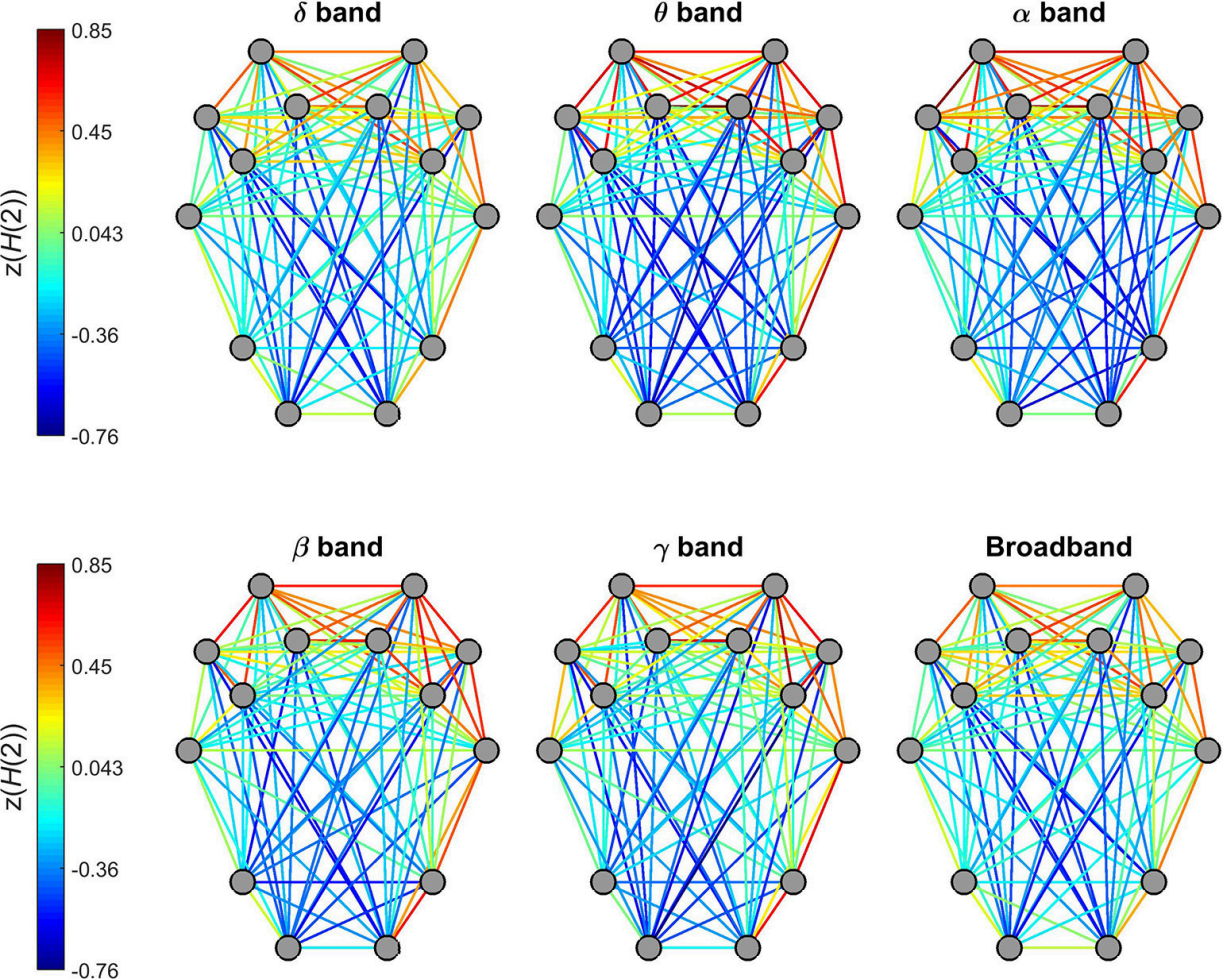
Topology of global long-range correlations in dynamic functional connections across the brain cortex. In all frequency bands as well as in broadband EEG, connections between frontal and prefrontal regions exhibit higher, while occipito-frontal connections lower *H*(2) values.

*H*(2) of individual connections showed a characteristic topological distribution in all frequency bands as well as broadband EEG data (Figure 5). In that spatially proximal functional connections between ROIs expressed higher, while those between distant regions showed lower *H*(2) values. This pattern could be observed most prominently in connections between regions of the frontal and pre-frontal cortex that had the highest z(*H*(2)) values, while in general connections linking regions of the occipital and parietal cortex to those of the frontal- and pre-frontal cortex had the lowest z(*H*(2)) values. Also, nearby connections linking contralateral regions in the frontal and pre-frontal areas expressed higher long-range correlation, however this did not hold for connections linking areas of the occipital and parietal cortex to those of the contralateral hemisphere (Figure 5).

#### Degree of multifractality–Δ*H*15

**Figure 6:**
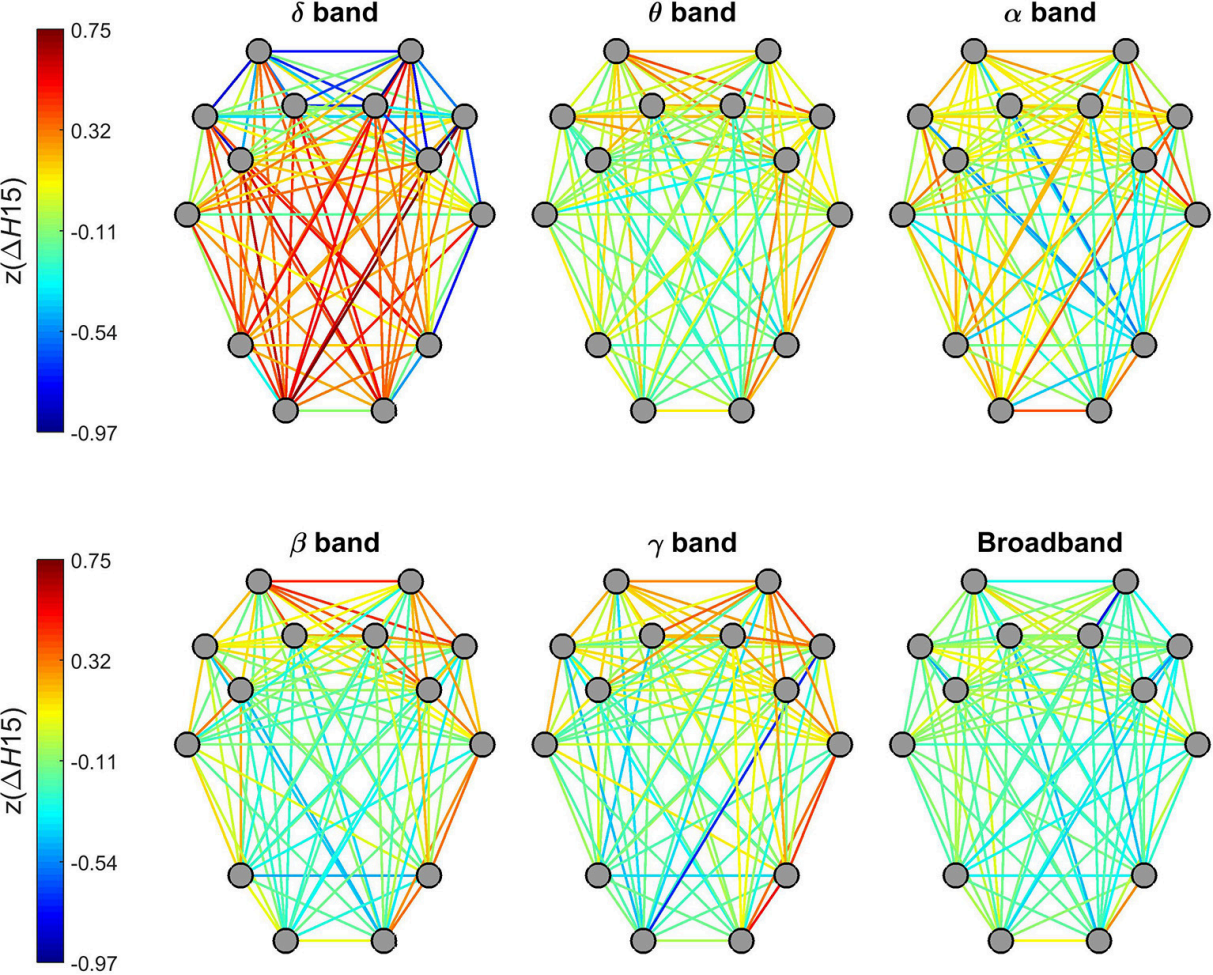
Topology of the degree of multifractality in dynamic functional connections across the brain cortex. Connections of the delta band showed a markedly different topology from the rest of the frequency bands. Also, no particular topological pattern was apparent in connections estimated on broadband EEG.

Topology of Δ*H*15 of individual connections in the delta band were markedly different from those of other frequency bands (Figure 6). While dynamic connections in the theta, alpha, beta and gamma bands resembled a topology similar to that observed for *H*(2) (with higher values for short- and lower-values for long-distance connections), a markedly opposite distribution appeared in the delta band. Within this range, stronger multifractality was found in connections linking the occipital and parietal regions to pre-frontal and frontal regions while lower Δ*H*15 values appeared in connections between nearby regions. Connections estimated on broadband EEG appeared to exhibit a homogenous distribution of Δ*H*15 without any particular topology (Figure 6).

#### Relationship between z(*H*(2)) and z(Δ*H*15)

**Figure 7:**
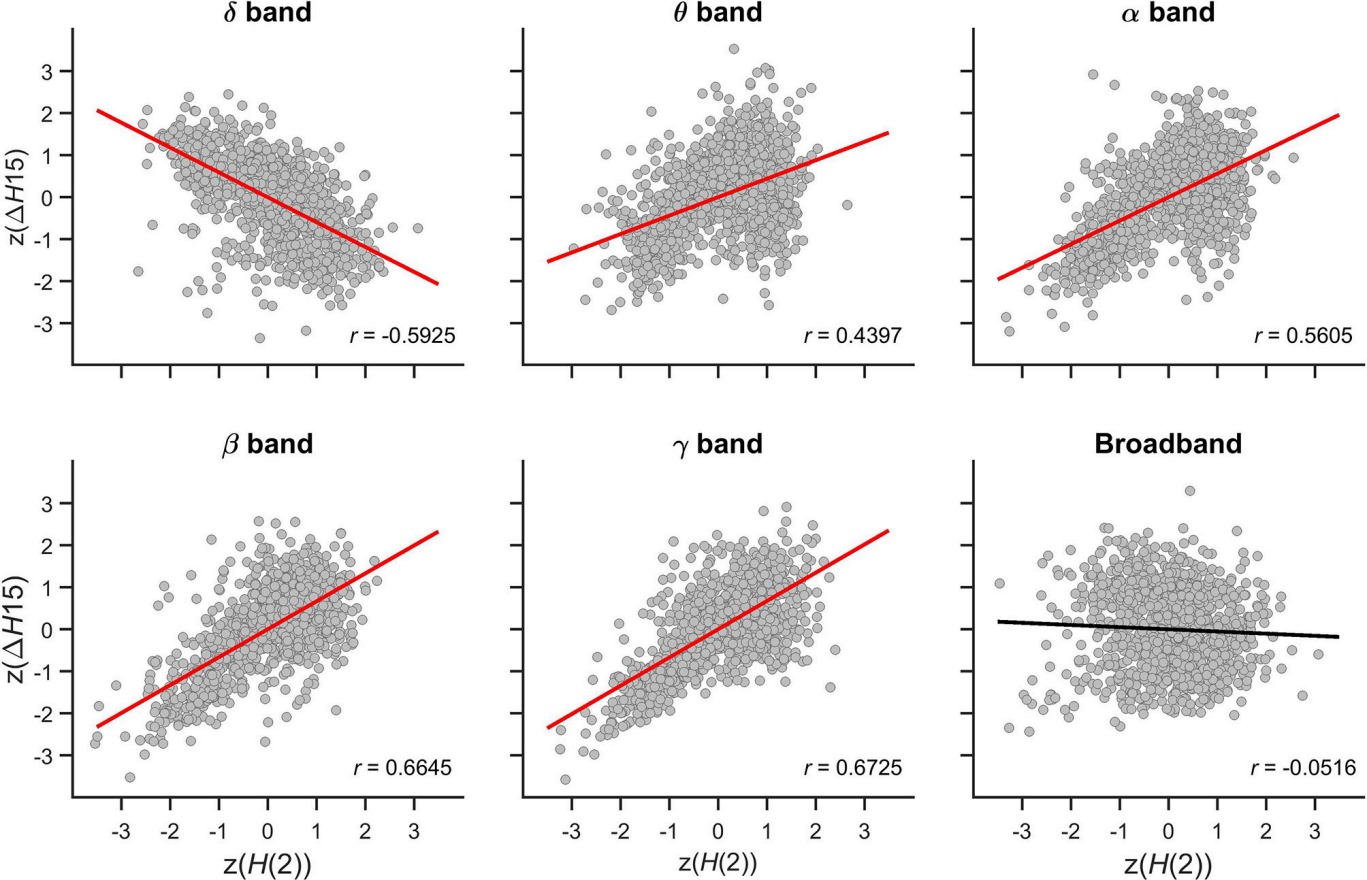
Cross-correlation between *z(H*(2)) and *z*(Δ*H*15) of individual connections. Panels are scatter plots of all connections (14^*^13/2) of all subjects (48). The Pearson correlation coefficient (*r*) is displayed in the lower right corner of each panel. Significant correlation is indicated by red regression line, while the black regression line for broadband EEG indicates that the correlation was indistinguishable from spatial surrogates.

The relationship between the multifractal properties of dynamic functional connections was captured in the Pearson correlation coefficient between z(*H*(2)) and z(Δ*H*15) values with their scatter plots shown in Figure 7. In the theta, alpha, beta, and gamma bands, significant positive correlations were found indicating that connections with higher long-term autocorrelation was associated with higher degree of multifractality. The opposite topology of z(*H*(2)) and z(Δ*H*15) in the delta band however resulted in a strong negative correlation as connections with high *H*(2) were associated with lower Δ*H*15 values. In broadband EEG no correlation was found concomitant with the absence of topology in Δ*H*15 of individual connections (Figure 5).

## Discussion

### Multifractal nature of global DFC

In this study, we reported that dynamic functional connectivity of the brain—as reconstructed from 14 channel whole-head EEG measurements and captured with dynamic graph theoretical analysis—fluctuates according to multifractal dynamics. Surrogate data tests proved that in majority, this temporal structuring was of true multifractal nature in all frequency bands as well as in broadband EEG. We also identified several significant differences in the MF characteristics of global DFC related to network measure, gender, and state.

We found that the degree of long-range temporal correlation could be attributed to specific topological aspects of the dynamic functional networks in that *H*_*C*_(2) was found significantly lower than *H*_*D*_(2) and *H*_*E*_(2). This pattern was present almost universally in all frequency bands as well as in broadband EEG data. A very similar pattern regarding the *H*(2) of the same dynamic graph theoretical measures (D, C, and E) was reported previously in Racz et al. (2018), where multifractal nature of DFC in the pre-frontal cortex was investigated using fNIRS imaging. Findings of the present study and those of Racz et al. (2018) suggest therefore that this pattern is a genuine feature of FC dynamics as it can be captured in different imaging modalities across a broad range of spatio-temporal scales. In Δ*H*15, significant differences related to network measure appeared prominently only in the delta band, where Δ*H*_*C*_15 was significantly lower than Δ*H*_*D*_15 and Δ*H*_*E*_15. These findings are also consistent with the similar results of Racz et al. (2018), where Δ*H*15 showed comparable differences regarding the applied network measures (Δ*H*_*D*_15 > Δ*H*_*C*_15 < Δ*H*_*E*_15). As in this study, this measure-related pattern was found only in the delta band with basically no significant measure-related differences in higher frequency bands. As Racz et al. (2018) investigated spontaneous brain activity in the 0.01–0.1 Hz range, these results may apply for lower-frequency brain activity, only.

Multifractal time series analysis characterizes the time-dependent scaling in a temporal process, that can emerge from intermittent periods with high variance (Ihlen and Vereijken, 2010). In this study, employing three graph theoretical measures–each capturing different aspects of network structure and topology–all were shown fluctuating in a multifractal manner, although to a different extent. The true multifractal scaling of all three network topological measures indicates that their scaling is in fact a local, time-dependent property. This implies that the temporal evolution of dynamic resting-state connection networks was interspersed with short periods of high variance in their segregation and integration, suggesting that these reorganization events leave their impact on localized and global information transfer alike (as captured in C and E, respectively). Moreover, the differences observed in the *H*(2) and Δ*H*15 values of these measures—at least in the delta band—imply that local and global information processing is affected differently: the lower Δ*H*_C_15 suggesting a more “balanced” temporal structuring of localized activity, while global network integration (related to fast information transfer between distant network sites) and overall “wiring cost” is associated with larger variability in scaling.

The scale-free nature of global DFC has been demonstrated earlier by Stam and de Bruin (2004). In that study, DFC was captured in global synchronization of EEG signals (as acquired by averaging SL time series) and fractal dynamics were characterized by the monofractal Hurst exponent estimated with Detrended Fluctuation Analysis. The authors found an increased Hurst exponent in eyes closed condition in the alpha and beta bands (Stam and de Bruin, 2004). As weighted Density is in fact the averaged connectivity strength (captured in SL), our current observations regarding the same difference–i.e., increased *H*(2) in the alpha and beta bands–are in agreement with those of Stam and de Bruin (2004) thereby extending their validity to Clustering Coefficient and Efficiency. The transition from eyes closed to eyes open state is often associated with desynchronization in cortical activity [Berger (1929), for a review see Barry et al. (2007)], with lower *H*(2) values reflecting on the dominance of rapid fluctuations (Stam and de Bruin, 2004). The lower *H*(2) values found in EO condition for all three network measures may indeed reflect the large-scale network reorganization affecting both local and global information flow (Rubinov and Sporns, 2010; Preti et al., 2017).

In a more recent study, Van de Ville et al. (2010) reported that EEG microstates also exhibit scale-free dynamics. Nevertheless, the authors did not find any difference in the scaling between various statistical moments as EEG microstates were found fluctuating in a strictly monofractal manner. However, this absence of MF dynamics could be the consequence of the applied analysis method. Specifically, it has been shown previously, that brain electrical activity fluctuates between only four different microstates in resting state (Lehmann et al., 1998). Van de Ville et al. (2010) used bipartitioning between these four microstates to capture microstate transitions, and confirmed fractal dynamics in all possible bipartitioning scenarios independently from the applied partition itself (Van de Ville et al., 2010). The random walk time series analyzed by Van de Ville et al. (2010) were acquired by cumulatively summing the bipartition label sequences (consisting of −1 and +1 values) of the EEG microstates. These are reminiscent of the sign time series obtained from an increment series (Ashkenazy et al., 2001, 2003), that can be obtained as the sign sequence of the local derivatives of a fluctuating signal. Sign time series were indeed shown to be related to the monofractal character (Gomez-Extremera et al., 2016), while scaling in the magnitude time series acquired as the absolute value sequence of local derivatives was reported to be related to multifractality of a dynamic process (Gomez-Extremera et al., 2016; Bernaola-Galvan et al., 2017). Our results show that dynamic graph theoretical analysis can capture the dynamics of FC in a more detailed fashion than EEG microstates, allowing for the unfolding of finer temporal structuring such as multifractal scaling.

Gender-related differences in DFC has also been reported previously (Yaesoubi et al., 2015a,b). Yaesoubi et al. (2015b) investigated the simultaneous occupation of different FC states (i.e., state combinations termed as “combo states”) and showed that males occupy a larger fraction of all possible combo states than females. Since spatio-temporally overlapping events were suggested as a possible origin of multifractal dynamics in a complex system (Lima et al., 2017), this may indeed be the underlying reason of the higher degree of multifractality of DFC observed in males in the alpha band. We also found higher autocorrelation i.e., higher *H*(2) values in male subjects in the delta band. It is well-known, that functional connectivity correlates well with structural (anatomical) connectivity (SC) (Greicius et al., 2009; Honey et al., 2009) even when investigated on multiple time scales (Honey et al., 2007). Our present findings showed that in some cases gender can have an influence on FC dynamics, which could well be–at least in part–to gender-related differences in brain anatomical connectivity (Ingalhalikar et al., 2014).

Finally, 1/*f* ^β^ i.e., scale-free neurodynamics were suggested in numerous studies as an indication of an underlying self-organized critical state of the brain (Linkenkaer-Hansen et al., 2001; Stam and de Bruin, 2004; Stam, 2005; Kitzbichler et al., 2009; Chialvo, 2010; Van de Ville et al., 2010; Tagliazucchi et al., 2012a; Racz et al., 2018). Moreover, it has been reported that not only mono- but indeed multifractal scaling could also occur in a three dimensional system at a critical state (Lima et al., 2017). The concept of self-organized criticality (SOC) as introduced by Bak et al. (1987) refers to the intrinsic property of a dynamic system constantly approaching a critical state without the fine tuning of an external “control parameter.” A critical state—where local perturbations are allowed to dissipate across all spatial and temporal scales in the system—usually appears near (first- or secondorder) phase transitions. Therefore, SOC is often considered as an ideal state of the brain in which fast adaptation to changes in the external or internal environment can be effectively achieved by rapid large-scale reorganization (Van de Ville et al., 2010). The scale-free–and indeed, multifractal–nature of the DFC measures reported in this study may therefore be considered evidencing yet another implication of the self-organized critical nature of resting-state brain activity.

### Multifractal dynamics of individual functional connections

We showed that DFC networks exhibited multifractal dynamics not only in their global graph theoretical parameters, but in their dynamic functional connections, too, as captured in the varying connectivity strengths between the nodes. This property was most prevalent in the delta, theta and alpha band connections, while slightly lower fraction of beta and gamma band as well as broadband EEG connections was proven as true multifractals.

Given the moderate spatial resolution in this study, instead of focusing on individual differences between inter-regional connections, we rather investigated if dynamic functional connections showed any global topology in their *H*(2) and Δ*H*15 values and if there was a relationship between them. In all frequency bands as well as in broadband EEG data we found a characteristic topology in the long-term autocorrelation of FC strength, as short-range connections between the frontal and pre-frontal areas tend to have higher *H*(2) values than long-range connections linking the same regions to the occipital and parietal cortex. A very similar topology regarding the degree of multifractality captured in Δ*H*15 was found in the theta, alpha, beta, and gamma bands. On the contrary, the topological pattern was the opposite in the delta band: long-range connections linking occipital regions with mostly the frontal and pre-frontal regions showed higher Δ*H*15 values than those linking nearby regions prominently again the frontal and pre-frontal areas. This inverse relationship in the delta band could be captured in a strong negative correlation between *H*(2) and Δ*H*15 values. However, in the higher frequency bands the topology of Δ*H*15 values were similar to those of *H*(2) thus resulting in strong positive correlation between the two. No correlation was found between the two MF measures in broadband EEG connections. It should be emphasized that a relationship between *H*(2) and Δ*H*15 is indeed non-trivial, as these measures capture two separate properties of the multifractal spectrum (i.e., spectral center and spectral width) (Theiler, 1990; Kantelhardt et al., 2002; Kantelhardt, 2009; Mukli et al., 2015).

It has been shown in dynamic processes, that monofractal scaling [captured in *H*(2) of the scaling function or the center of the multifractal spectrum] is attributed to linear properties, while the degree of multifractality [as assessed equivalently either by Δ*H*15 or the multifractal spectrum width] correlates well with the degree of non-linearity (Gomez-Extremera et al., 2016; Bernaola-Galvan et al., 2017). In addition non-linearity in a power-law correlated signal can also be well estimated by the long-term autocorrelation of its magnitude time series (Ashkenazy et al., 2003; Schmitt et al., 2009). Multifractality often emerges from the presence of intermittent periods of higher variance (Ihlen and Vereijken, 2010). Accordingly, from the topological pattern observed in broadband EEG and in the lower frequency range (i.e., delta band), it is apparent that connections between nearby regions express a lower degree of non-linearity and they are more linearly autocorrelated (however in most cases still non-linear as well), while on the contrary, the opposite is true for long-distance connections where linear autocorrelation is weaker and the dynamics appear more non-linear. Therefore our results are in good agreement with—and presumably reflect the same phenomenon as—previous findings demonstrating the non-linear nature of neuronal synchronization (Stam et al., 2003) and intermittent periods of high synchronization between neuronal cell assemblies (Friston, 2000). Also, this is in line with the fact that brain activity is generally scale-free on the large-scale level while becoming more synchronized when observed smaller spatial scales (Buzsaki, 2006).

Dynamic synchronization levels mainly capture the joint activity of neuron populations of the regions of interest. The fractal and multifractal nature of individual connections therefore may also indicate that a possible critical state of brain activity may not only be present on a global level, but also on smaller spatial scales, too. In a SOC simulation using a modified version of the classic sand pile model, Mukli et al. (2018) investigated the effect of the size and connection density of the cellular automata on the multifractal properties of their dynamics, which was captured in the total number of sand grains resident in the system at a given moment. Their results demonstrated, that with (independently) increasing system size or connection density, both *H*(2) and Δ*H*15 of the system dynamics increased (Mukli et al., 2018). This phenomenon is very reminiscent of the positive correlation found in this study between *H*(2) and Δ*H*15 of individual connections in the higher frequency bands. Therefore, higher *H*(2) and Δ*H*15 values of a dynamic connection may well reflect that the joint activity between the two regions involved larger and/or more densely interconnected neuronal populations. Also, the frequency range of network oscillations is constrained by the networks size because most neuronal connections are local and thus yield high-frequency oscillations (Braitenberg and Schüz, 2013). Accordingly, slower oscillations can only be produced when larger neuronal cell assemblies are involved (Buzsaki and Draguhn, 2004). Higher *H*(2) values indicate the dominance of slow fluctuations thus suggesting the involvement of large neural populations. These considerations are well in line with the results of this study regarding that the highest *H*(2) and Δ*H*15 values were found in connections of the frontal and prefrontal cortex; regions that are both anatomically and functionally well connected and form a high-level association cortex with diverse functionality (Kandel, 2013). On the contrary, lower values were found between both anatomically and functionally distant regions.

Nevertheless the inverse relationship between *H*(2) and Δ*H*15 found in the delta band raises some questions. Delta band activity is not prominent in awake state during physiological conditions, however several studies demonstrated the critical nature of brain dynamics during sleep stages (Lo et al., 2002, 2004, 2013). It also has been shown recently (using an alternate DFC approach termed time delay stability, TDS), that different sleep stages–that are known to be dominated by specific EEG frequency bands, as α oscillations are most prominent during quiet wake and rapid eye movement, while θ and δ fluctuations usually characterize light- and deep sleep, respectively–exhibit FC topologies characteristic to sleep stage and frequency band as well (Bartsch et al., 2015; Liu et al., 2015). Therefore, sleep would probably be a better setting for further investigating the multifractal and critical nature of delta band connections—and their relation to other frequency bands—that is evidently beyond the scope of this present study.

### Limitations, future perspectives

Multifractal analysis could reveal relevant information at the global (network) and the local (connection) levels, that otherwise may remain hidden in static and also in most dynamic functional connectivity approaches. MF analysis of DFC carries potential for future applications both in basic science and clinical fields, however one has to consider some methodological difficulties with this approach. For a reliable numerical estimation of fractal and/or multifractal parameters, a signal length of at least a few thousand data points is desired, as well as long-enough measurement time and high temporal resolution so that the signal could represent a sufficiently broad range of temporal scales (Eke et al., 2002; Ihlen, 2013). While these prerequisites can be readily met with imaging modalities such as EEG, fNIRS, or MEG, still major drawbacks remain owing to their lower spatial resolution, their lack of exact source localization and the fact that these techniques cannot access subcortical regions. These limitations can be partly overcome by using fMRI—with spatial normalization even allowing for exact comparison between separate studies—however at the expense of lower temporal resolution and limited signal length, both considerably affecting the applicability of multifractal analysis (Eke et al., 2012). As these methods–e.g., EEG and fMRI–can provide complementary information, the importance of simultaneous EEG-fMRI (and/or fNIRS-fMRI) measurements is indeed crucial in revealing the relationship between EEG-DFC and fMRI-DFC so that DFC could be investigated with high temporal resolution and exact spatial localization, alike.

Although in this study the whole brain cortex is sampled, the spatial resolution is still fairly limited. Using a higher spatial resolution method would not only benefit from a more detailed sampling of brain activity, but would also allow for calculating more complex network measures—i.e., those related with modularity, centrality or network motifs being more complex than triangles—that could reveal even more details on functional brain organization. Also, from Table 1 it is apparent, that gamma band activity may not be well represented in the state space during SL calculation. This is a limitation brought about by the sampling frequency that calls for greater caution when results regarding the gamma band are evaluated.

DFC analyses carry great potentials not only in basic research leading to a better understanding of brain functions, but also in the clinical field, as several studies already demonstrated their applicability in neuropsychiatric diseases such as schizophrenia (SZ) (Sakoglu et al., 2010; Calhoun et al., 2014; Damaraju et al., 2014) or autism (Price et al., 2014). Thus dynamic graph theoretical analyses similar to the one presented in this study could prove a useful and potentially powerful tool when investigating DFC in clinical settings. When analyzing the DFC of the default mode network with fMRI imaging in SZ patients, the same dynamic graph theoretical measures as used in this study were found to be fluctuating around a lower average value than in healthy controls (Du et al., 2016). Moreover in a whole brain DFC study on SZ patients, in addition to the same results it was also demonstrated that D(*t*), C(*t*), and E(*t*) showed less variance than in healthy controls (Yu et al., 2015). Results of the present study clearly show, that the standard deviation (i.e., the square root of variance) of graph theory metrics depends on the observation scale and that second order statistics alone are insufficient to fully characterize the network dynamics. Multifractal analysis however is suitable to capture such features, therefore may serve as a more sensitive tool in distinguishing physiological states from pathological conditions based on their dynamic graph theoretical measures.

Finally, in a recently established field of biological systems science termed network physiology (Bashan et al., 2012; Ivanov and Bartsch, 2014), the dynamic interactions of local neural activity with several other physiological subsystems (e.g., the cardiac and respiratory system) were analyzed (Bartsch and Ivanov, 2014; Bartsch et al., 2015; Lin et al., 2016). These studies showed that during different physiological states such as sleep stages, the interactions between the elements of this physiological network change significantly. As functional connectivity was also shown to alter during different physiological conditions (Horovitz et al., 2009; Liu et al., 2015; Racz et al., 2017), a dynamic graph theoretical approach may contribute to this emerging field by providing a way for capturing coordinated states of neural activity so that its interactions with other functions of the human body could be further analyzed.

## Conclusions

In this study, we showed that dynamic global functional connectivity of the brain—when investigated by EEG mapping and captured in dynamic graph theoretical measures—fluctuates according to a multifractal temporal pattern. Our results revealed that several network topological aspects exhibit different characteristics. Moreover, the dynamic functional connections assembling these networks showed multifractal dynamics themselves. We found a characteristic topology in both mono- and multi-fractal measures with a positive correlation between them in the higher frequency bands, while anticorrelation in the delta band. Our results suggest that multifractality is indeed a fundamental property of both global and local (i.e., individual) DFC with specific global and local attributes to network topology and anatomical localization, respectively. Our findings are in support of a possible self-organized critical nature of resting-state brain activity. We propose that multifractal analysis can provide a more detailed description of global and local connectivity dynamics than most methods applied in the field, and it could serve as a valuable tool for a better characterization of healthy and pathological brain functions.

## Author contributions

FR carried out the EEG measurements, developed the analysis framework, performed the analysis on measured data, and wrote the manuscript. OS helped carrying out the measurements and did the data pre-processing. PM helped conducting the measurements and writing the manuscript. AE helped developing and writing the manuscript and provided conceptual guidance in the study.

### Conflict of interest statement

The authors declare that the research was conducted in the absence of any commercial or financial relationships that could be construed as a potential conflict of interest. The handling editor is currently co-organizing a Research Topic with one of the authors, AE and confirms the absence of any other collaboration.
